# A Novel c-Mesenchymal-Epithelial Transition Factor Intergenic Fusion Response to Crizotinib in a Chinese Patient With Lung Adenocarcinoma: A Case Report

**DOI:** 10.3389/fonc.2021.727662

**Published:** 2021-10-28

**Authors:** Hongge Liang, Dexun Zhou, Lin Dai, Moqin Zhang, Zhancheng Gao, Xinlin Mu

**Affiliations:** ^1^ Department of Respiratory and Critical Care Medicine, Peking University People’s Hospital, Beijing, China; ^2^ Department of Pathology, Peking University People’s Hospital, Beijing, China

**Keywords:** lung adenocarcinoma, MET intergenic fusion, crizotinib, partial response, interstitial lung disease

## Abstract

**Background:**

The c-mesenchymal–epithelial transition factor (C-MET) is an oncogene encoding a tyrosine kinase receptor that plays an important role in tumor growth and metastasis. The National Comprehensive Cancer Network (NCCN) guidelines have approved carbatinib/crizotinib for advanced non-small cell lung cancer (NSCLC) patients with MET exon 14 skipping.

**Methods:**

In June 2020, the Department of Respiratory and Critical Care Medicine of Peking University People’s Hospital admitted a 72-year-old male patient with lung adenocarcinoma (LADC) with a history of interstitial lung disease secondary to antineutrophil cytoplasmic antibody-associated vasculitis. Genetic examination by next-generation sequencing showed an intergenic fusion of MET, and crizotinib was administered on August 14, 2020. Follow-up showed that tumor volume was significantly reduced. However, crizotinib was discontinued in November 2020 because of the patient’s worsening interstitial lung disease, and CT scans showed continued partial response (PR) for 5 months. In April 2021, right lower lobe mass progressed, and disease progression was considered.

**Conclusion:**

This was the first case of a patient with LADC with MET intergenic fusion who significantly benefited from crizotinib. Even after crizotinib was discontinued for 5 months, the patient continued exhibiting PR, suggesting that MET intergenic fusion may have carcinogenic activity in LADC and was sensitive to crizotinib.

## Introduction

Lung cancer is the main cause of cancer-related deaths worldwide, with non-small cell lung cancer (NSCLC) accounting for approximately 85% of the mortality. NSCLC diagnosis mainly depends on pathology, and most patients are often diagnosed at late stage with poor prognosis. The emergence of targeted therapy has greatly improved the progression-free survival (PFS) and overall survival of patients with advanced NSCLC. Therefore, routine genetic testing is required for patients with NSCLC. The c-mesenchymal–epithelial transition factor (C-MET) gene is an oncogene locating on chromosome 7q21-31 and encoding a tyrosine kinase receptor (1). MET alterations include point mutations, amplification, fusion, and protein overexpression, which can lead to the proliferation, invasion, and metastasis of tumor cells by downstream signaling pathways such as RAS-extracellular signal-regulated kinase/mitogen-activated protein kinase (RAS-ERK MAPK), signal transducers and activators of transduction-3 (STAT3), and phosphatidylinositol 3-kinase/protein kinase B (PI3K/AKT) (2–4). Previous studies (5) have shown some antitumor activity of MET inhibitors in NSCLC, indicating that MET is a promising therapeutic target for patients with advanced NSCLC.

Crizotinib is a small-molecule tyrosinase inhibitor that effectively inhibits MET (high-level MET amplification or MET exon 14 skipping mutation), anaplastic lymphoma kinase (ALK), and reactive oxygen species (ROS) proto-oncogene 1 (ROS1) (6–10). The National Comprehensive Cancer Network (NCCN) guidelines have approved crizotinib for advanced NSCLC patients with ALK/ROS1 rearrangement. An expansion cohort of the PROFILE 1001 (8) study evaluated the antitumor activity and safety of crizotinib in 69 patients with advanced NSCLC harboring MET exon 14 skipping mutations. The results showed that objective response rate (ORR) was 32% (95% CI, 21%–45%) among 65 response-evaluable patients. The median duration of response was 9.1 months (95% CI, 6.4–12.7 months). The median PFS was 7.3 months (95% CI, 5.4–9.1 months). Therefore, NCCN guidelines approved crizotinib as a first-line therapy or subsequent therapy option (category 2A; useful in certain circumstances) for patients with metastatic NSCLC harboring MET exon 14 skipping mutations. MET fusions are rare in LADC, and the standard treatment for these patients has not been determined. To our knowledge, there have been no reports of LADC with MET intergenic fusion. Here, we present a case of lung adenocarcinoma (LADC) with a novel MET intergenic fusion response to crizotinib treatment.

## Case Description

**Figure 1:**
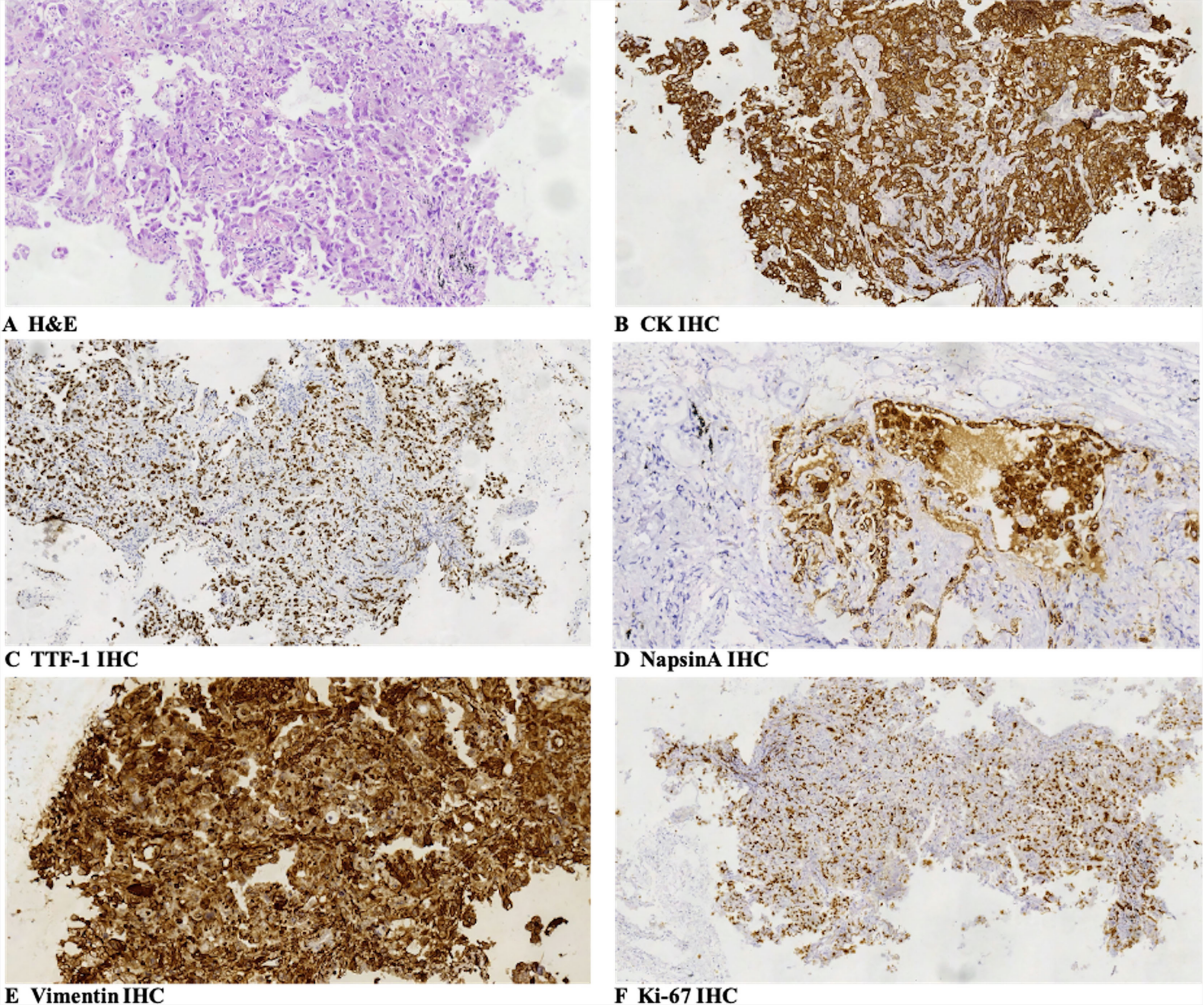
Pathologic findings: **(A)** the large-sized epithelioid tumor cells were arranged in a nest-like pattern, and they had wide cytoplasm with obvious atypia, large and clear cell nuclear, and nuclear fission. The pathologic diagnosis of the biopsy specimens was poorly differentiated lung adenocarcinoma (H&E, SP ×200). **(B–F)** Immunohistochemical staining showed that tumor cells were positive for CK (**B**, SP ×200), TTF-1 (focal positive, **C**, SP ×100), NapsinA (**D**, SP ×200), and Vimentin (**E**, SP ×200), and Ki-67 was 60% (**F**, SP ×100). SP, streptavidin–peroxidase.

**Figure 2:**
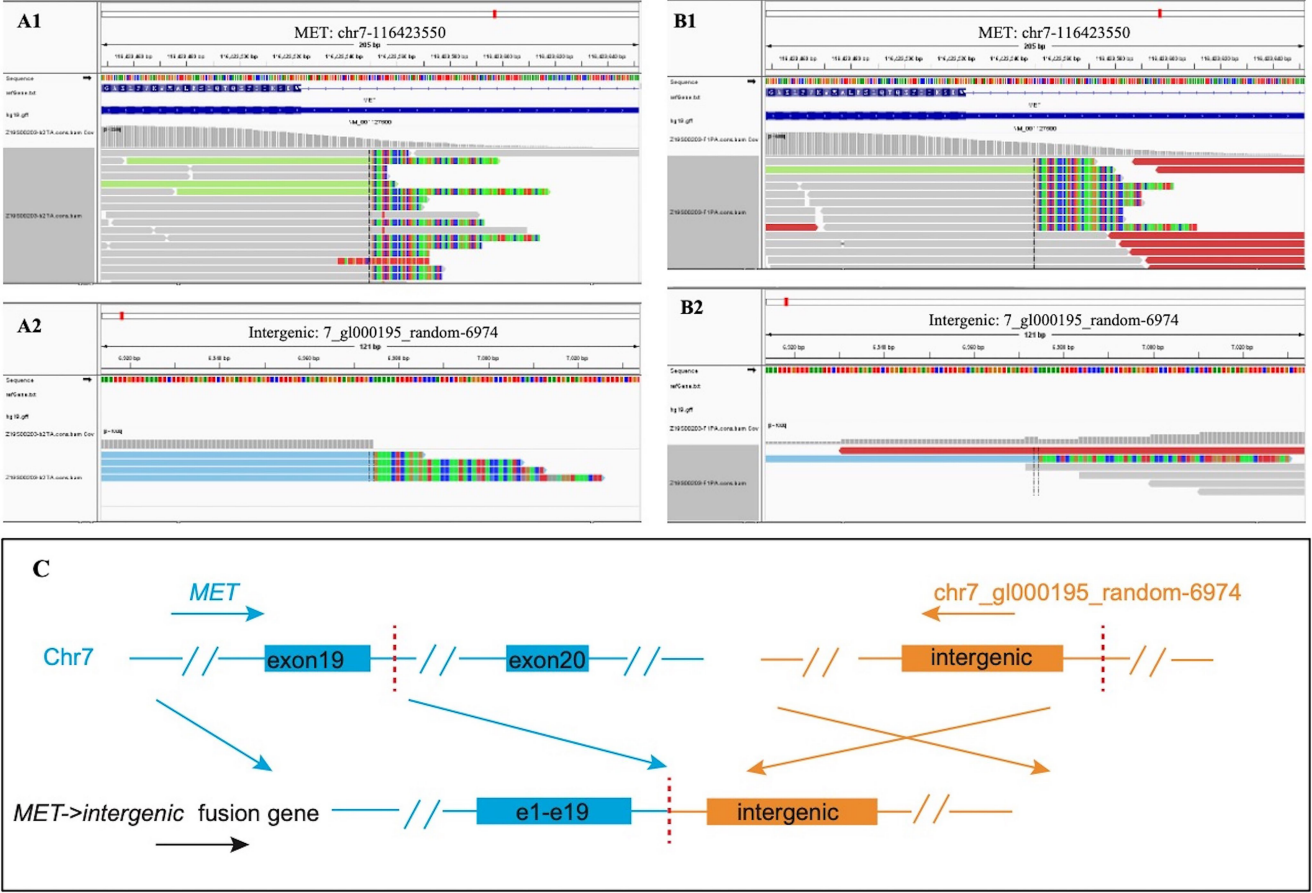
Next-generation sequencing revealed MET intergenic fusion. Mapping of fusion junction sequence revealed the fusion of random sequences to MET gene. The breakpoints were located on chr7_gl000195_random: 6974 and chr7:116423550 in intron 19 of the MET gene, and the sequence of MET is reversed. **(A)** DNA sequencing reads indicating fusion region by genomics Viewer software in tissue. **(B)** DNA sequencing reads indicating fusion region by genomics Viewer software in plasma. **(C)** The breakpoints detected in the fusion by targeted next-generation sequencing (NGS) and the alterations in gene caused by this fusion.

**Figure 3:**
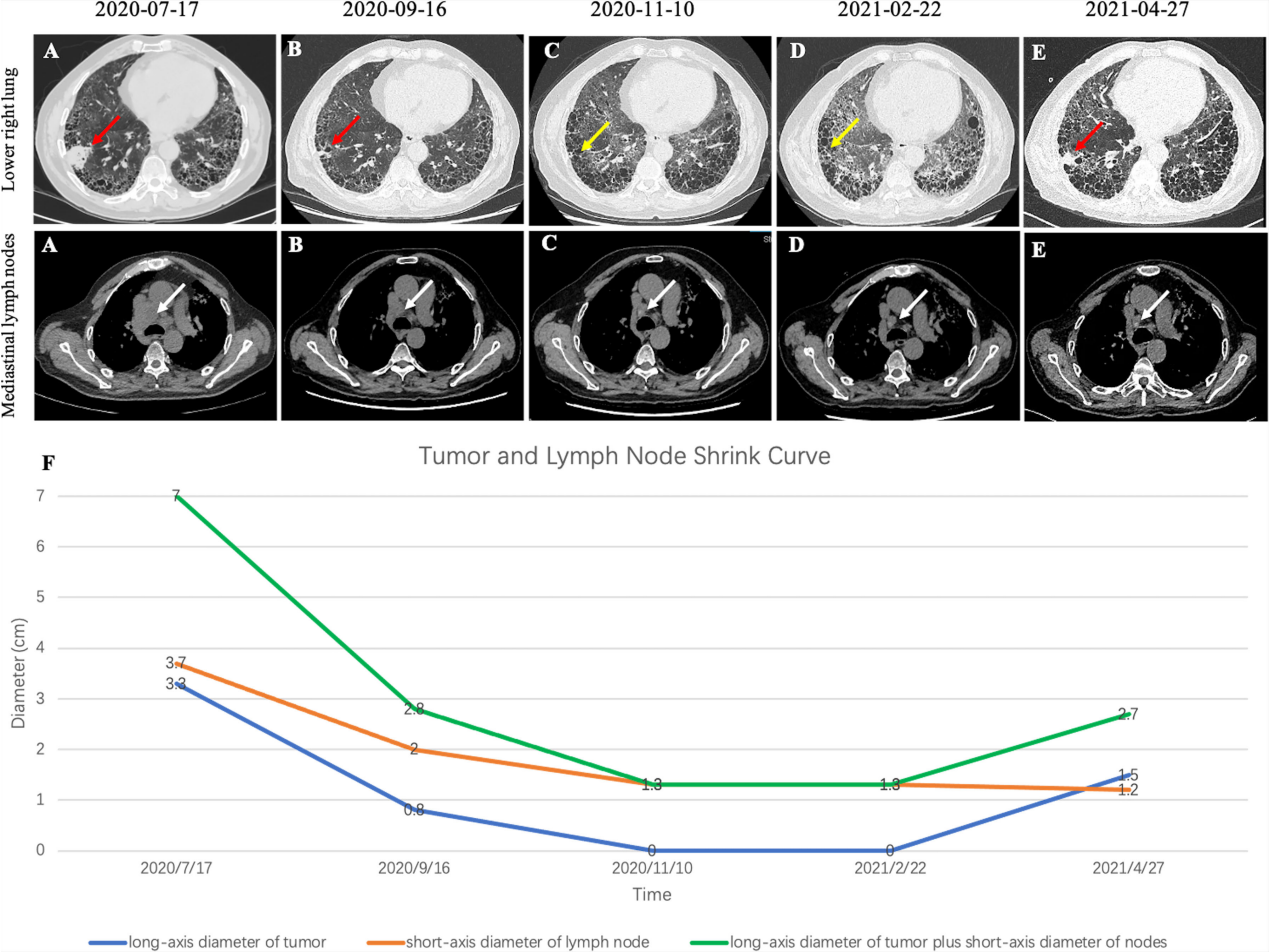
**(A)** CT image before crizotinib treatment. **(B)** CT image at 1 month after crizotinib treatment beginning. **(C)** CT image at 3 months after start of crizotinib treatment. **(D)** CT image at 3 months after discontinuing treatment. **(E)** CT image at 5 months after discontinuing treatment. **(F)** Tumor and lymph node shrink curve. Note that red arrows indicate the tumor. Yellow arrows indicate that the primary tumors almost disappeared. White arrows indicate the lymph nodes.

**Figure 4:**
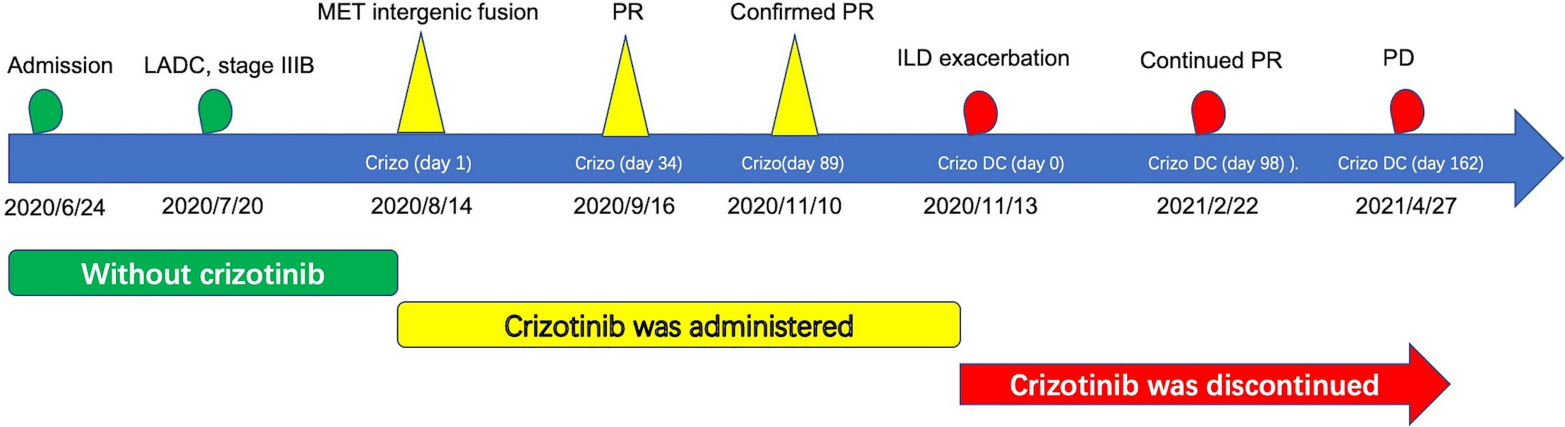
Historical and current information from this episode of care organized as timeline. LADC, lung adenocarcinoma; MET, mesenchymal–epithelial transition factor; PR, partial response; PD, progressive disease; ILD, interstitial lung disease; Crizo, crizotinib; Crizo DC, crizotinib was discontinued.

The patient was a 72-year-old male smoker with advanced NSCLC. He had a history of interstitial lung disease (ILD) secondary to antineutrophil cytoplasmic antibody-associated vasculitis and no other major medical problems. The patient presented in June 2020 with a 4-month history of dyspnea. Chest CT scans revealed a right lower lobe mass (long-axis diameter was 3.7 cm) and bilateral enlarged mediastinal/hilar lymph nodes. In July 2020, he underwent CT-guided lung biopsy and was pathologically diagnosed with poorly differentiated LADC (**Figure 1**). The enhanced MRI of the brain, bone scan, and contrast-enhanced CT showed no extrapulmonary metastases. Therefore, the patient was diagnosed with stage IIIB LADC (pT1N3M0) according to the eighth edition of TNM staging. Genetic examination by next-generation sequencing further showed an intergenic fusion of MET (**Figure 2**). The patient lost his surgery opportunity due to local advanced stage and history of ILD. Therefore, on August 14, 2020, he received crizotinib (250 mg twice a day). Routine CT scans performed after 1 and 3 months of therapy showed partial response (PR) of the lung tumor and lymph metastases (**Figure 3**). In November 2020, the patient’s ILD worsened. The laboratory data revealed negative myeloperoxidase (MPO) antibodies with a titer of 0.00 (normal value: <20 AU/ml) and negative proteinase 3 (PR3) antibodies with a titer of 5.53 (normal value: <20 IU/ml); thus, crizotinib was not ruled out as a cause of ILD exacerbation. Therefore, crizotinib was discontinued, and the patient did not receive any antitumor therapy. In February 2021, CT scans showed continued PR after 5 months after crizotinib therapy was discontinued (**Figure 3**). However, the right lower lobe mass progressed in April 2021 (**Figure 3**), and disease progression was considered. **Figure 4** showed the timeline with relevant data from the episode of care.

## Discussion

To our knowledge, this was the first case of a patient with LADC with MET intergenic fusion who significantly benefited from crizotinib.

MET fusion genes are composed of MET lacking the regulatory para-membrane domain and have different N-terminal partners. These fusion proteins lead to the activation of the kinase domain and are involved in tumorigenesis and progression (5, 11). Previous case reports have shown that crizotinib was effective against patients with LADC with MET fusion. Cho et al. (12) reported a patient with LADC with (the kinesin family 5B gene) (KIF5B)-MET fusion who had dramatic tumor shrinkage and duration of response to crizotinib. Plenker et al. (13) showed that crizotinib was effective in both LADC patients with KIF5B-MET fusion and steroidogenic acute regulatory protein-related lipid transfer domain-3 N-terminal like (STARD3NL)-MET fusion. Davies et al. (14) described a case of LADC harboring human leukocyte antigen (HLA)-DRB1-MET fusion response to crizotinib. These case reports suggested that crizotinib was a promising agent for treating MET-rearranged LADC.

The present case described a novel MET fusion pattern, MET intergenic fusion, in LADC. According to the genome annotation of the regions containing the breakpoints, fusions are classified into three types (gene–gene, gene–intergenic, and intergenic–intergenic). Among these three types, the intergenic–intergenic fusion does not generate chimeric transcripts and upregulates a gene by a different promoter or enhancer on the upstream of the gene (15). However, the effect of intergenic fusion on the transcriptome is still unclear, and they were excluded from the majority of fusion analyses. We found that patients with LADC with MET intergenic fusion significantly benefited from crizotinib treatment. Even after crizotinib was discontinued for 5 months, the patient continued exhibiting PR, suggesting that MET intergenic fusion may have carcinogenic activity in LADC and was sensitive to crizotinib. This suggests that genetic testing should be routinely performed in patients with NSCLC, which is often limited in clinical practice by the patients’ affordability or insufficient specimen size. However, our findings are limited by the focus on one case only, and more cases are needed for further confirmation.

In conclusion, we found a case of LADC with novel MET intergenic fusion response to crizotinib, suggesting that this fusion may have carcinogenic activity in LADC. Further, gene sequencing should be routinely performed in patients with NSCLC.

## Data Availability Statement

The original contributions presented in the study are included in the article/supplementary materials. Further inquiries can be directed to the corresponding author.

## Ethics Statement

The studies involving human participants were reviewed and approved by the Institutional Ethics Committee of Peking University People’s Hospital. The patients/participants provided their written informed consent to participate in this study.

## Author Contributions

HL: resources, formal analysis, validation, and writing—original draft. DZ: resources. LD: resources and visualization. MZ: resources. ZG: resources. XM: conceptualization, formal analysis, supervision, and writing—review and editing. All authors contributed to the article and approved the submitted version.

## Funding

This study was supported by the CAPTRA-LUNG (Grant No. CAPTRALung2021001).

## Conflict of Interest

The authors declare that the research was conducted in the absence of any commercial or financial relationships that could be construed as a potential conflict of interest.

## Publisher’s Note

All claims expressed in this article are solely those of the authors and do not necessarily represent those of their affiliated organizations, or those of the publisher, the editors and the reviewers. Any product that may be evaluated in this article, or claim that may be made by its manufacturer, is not guaranteed or endorsed by the publisher.
